# Bio-Based Phase Change Materials for Sustainable Development

**DOI:** 10.3390/ma17194816

**Published:** 2024-09-30

**Authors:** Mehdi Zadshir, Byung-Wook Kim, Huiming Yin

**Affiliations:** 1 Department of Civil Engineering and Engineering Mechanics, Columbia University, 500 W 120th Street, New York, NY 10027, USA; m.zadshir@columbia.edu (M.Z.); yin@civil.columbia.edu (H.Y.); 2 PVT Clean Energy, 7 Industry Street, #5, Poughkeepsie, New York, NY 12603, USA

**Keywords:** phase change material, animal fat, plant oil, fatty acids, latent heat, melting temperature

## Abstract

The increasing global population has intensified the demand for energy and food, leading to significant greenhouse gas (GHG) emissions from both sectors. To mitigate these impacts and achieve Sustainable Development Goals (SDGs), passive thermal storage methods, particularly using phase change materials (PCMs), have become crucial for enhancing energy efficiency and reducing GHG emissions across various industries. This paper discusses the state of the art of bio-based phase change materials (bio-PCMs), derived from animal fats and plant oils as sustainable alternatives to traditional paraffin-based PCMs, while addressing the challenges of developing bio-PCMs with suitable phase change properties for practical applications. A comprehensive process is proposed to convert bacon fats to bio-PCMs, which offer advantages such as non-toxicity, availability, cost-effectiveness, and stability, aligning with multiple SDGs. The synthesis process involves hydrolysis to break down fat molecules obtained from the extracted lipid, followed by three additional independent processes to further tune the phase change properties of PCMs. The esterification significantly decreases the phase transition temperatures while slightly improving latent heat; the UV-crosslinking moderately raises both the phase transition temperature and latent heat; the crystallization remarkably increases the both. The future research and guidelines are discussed to develop the large scale manufacturing with cost effectiveness, to optimize synthesis process by multiscale modeling, and to improve thermal conductivity and latent heat capacities at the same time.

## 1. Introduction

The rapid growth of the global population has spurred an increased demand for energy and food, both of which contribute to greenhouse gases (GHGs) emissions [1]. According to the International Energy Agency (IEA), carbon dioxide (CO_2_) emissions from the global energy system reached a new record high of 37 Gt in 2022, reflecting a substantial rise in fossil fuel use over the past two decades [2,3]. Meanwhile, the food system has become a major source, emitting 18 Gt CO_2_ equivalent per year, with about one third coming from energy-related activities [4,5]. Particularly, meat-based products emit twice as much GHGs as plant-based foods over the past century [1]. By 2050, even though the global population is projected to reach 9.1 billion with the annual meat production of 470 Mt [6], it will be challenging to achieve the goal as GHGs need to be minimized and the global energy demand needs to be reduced by 40% to avoid catastrophic climate tipping points without exceeding 1.5 °C global warming [7,8]. If bio-materials can be used to reduce GHG emissions, it may create a new way to reach the carbon neutralization.

Given that such global issue requires technical as well as socioeconomic transformations [9], passive thermal storage methods have become significant in the industries and building thermal management, which are the main sources of GHG emissions for heating, cooling, and fuel transportation or energy transmission. They enhance energy efficiency and reduce GHG emissions, linking to the Sustainable Development Goals (SDGs) in Table 1 across various industries such as building, food, and medicine [10,11]. Phase change materials (PCMs), which absorb or release latent heat during the phase transitions, play an important role in passive thermal management for heating, cooling, and packaging applications with zero GHGs emission [12,13,14,15]. For example, PCMs have been used to reduce 8.03–10.95 ton CO_2_ equivalent per year per hectare by decreasing operation of root zone heating systems, which improve crop quality and productivity, thereby contributing to SDG 2 of ‘Zero Hunger’ and SDG 13 of ‘Climate Action’ [15,16]. Moreover, SDG 7 of ‘Affordable and Clean Energy’ can be addressed by incorporating passive thermal methods into active thermal systems for space cooling and by lowering cost of heat storage and transportation [11,17]. However, those approaches have used paraffin-based PCMs derived from crude oil, another major source of GHG emissions [18]. In this context, natural sources of animal fats and plant oils have emerged as promising candidates among sustainable PCMs due to the non-toxicity, availability, cost-effectiveness, and stability [19,20]. Since they can be obtained from food wastes, this approach could be also interpreted in terms of SDG 12 of ‘Responsible Consumption and Production’ by reducing food wastes for the passive thermal management [21].

Although bio-PCMs could contribute to SDGs and sustainable food system without using crude oil sources, challenges still exist to obtain viable PCMs with appropriate synthesis processes. Previous research has primarily worked on partial processes, investigating either the conversion from fatty acids to PCMs or from food sources to fatty acids [20,25,26]. Moreover, for sustainable bio-PCMs, animal fats and plant oils have been used from various sources as shown Table 2. As one kind of animal fats, pork fats were obtained from waste cooking fats of italian sausages, local market, or slaughterhouse [27,28,29]. Non-edible pig and chicken parts were gifted [30], and beef tallow was purchased from a market [31]. As plant oils, coconut oil, palm oil, and palm kernel fat were obtained from the Soya group, bakery, and Pacific interlink SDSBHD, respectively [32,33,34]. Although lithium salt of dihydroxystearic acid (DHSA) showed $\Delta H_{m}$ of 236 J/g, the corresponding $T_{m}$ was much higher than ambient temperature to be used for human thermal comfort [30], and overall $\Delta H_{m}$ have shown insufficient for practical use as PCMs due to the limited latent heat ranging from 18 to 58 J/g for thermal energy storage applications. In comparisons, paraffin-based commercial PCMs exhibited a wider range of latent heat from 80 to 260 J/g [35,36]. Therefore, further synthesis processes need to be applied to have more favorable latent heat in phase change.

In addition to the latent heat, thermal conductivity is another important physical quantity in thermal energy storage, as it determines efficiency of heat conduction with PCMs [34]. However, as shown in Table 2, thermal conductivity (κ) of bio-PCMs is about 0.1–0.2 W/mK and fatty acid components have similar ranges as well in Table 3. The inclusion of graphite into palm oil based PCM increased κ from 0.2 to 0.86 W/mK, whereas $\Delta H_{m}$ decreased from 74.35 to 41.13 J/g instead [34]. While it is considered as trade-off relationship between $\Delta H_{m}$ and κ of bio-PCMs, recently, adding hexagonal boron nitride (h-BN) of 2 wt.% into myristic acid and lauric acid exhibited $\Delta H_{m}$ = 197.47 J/g with κ = 0.23 W/mK and $\Delta H_{m}$ = 185.30 J/g with κ = 0.26 W/mK, respectively [37].

**Figure 1:**
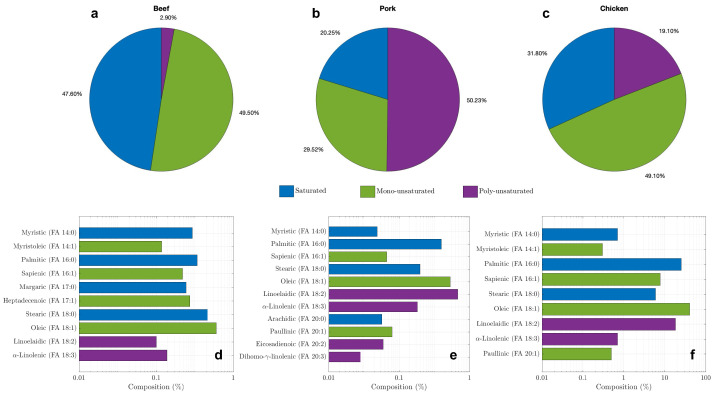
Compositions of animal fats: saturated (blue), mono-unsaturated (green), and poly-unsaturated (purple) fatty acids for beef (**a**), pork (**b**), and chicken (**c**); fatty acids breakdown for beef (**d**), pork (**e**), and chicken (**f**) [53,54,55].

As the obtained fatty acids can be typically analyzed by mass spectrometry [52], compositions of fatty acids from three kinds of animal sources, beef, pork, and chicken, are compared in Figure 1 [53,54,55]. Those animal fats are characterized in terms of saturated (blue), mono-unsaturated (green), and poly-unsaturated (purple) in Figure 1a–c, respectively. Overall, beef shows the highest fraction of saturated fatty acids and the lowest fraction of unsaturated fatty acids, which is the summation of mono-unsaturated and poly-unsaturated acids, whereas pork has the lowest fraction of saturated fatty acids. Each fatty acid composition is shown as name, C:D (carbon to double-bond) ratio, and fraction in Figure 1d–f. For instance, myristic acid (14:0) is saturated with 14 carbons and 0 double-bond, while oleic acid (18:1) is mono-unsaturated with 18 carbons to 1 double-bond (=) ratio, which is indicated with its chemical formula in Table 3 as well. Stearic acid (18:0) is the largest among saturated fatty acid in beef, and oleic acid (18:1) is the major unsaturated fatty acid in beef and chicken. Pork and chicken have the largest saturated fatty acid of palmitic (16:0), while pork has the major unsaturated fatty acid of linoelaidic (18:2). Fatty acids are made up of a hydrocarbon chain ending in a carboxylic acid group (COOH) as the corresponding chemical formula is shown in Table 3. The length of the hydrocarbon chain commonly varies from 8 to 24 carbons, and the chain typically is linear when it has an even number of carbons, unless double bonds are present, which cause the chain to become bent [56].

**Figure 2:**
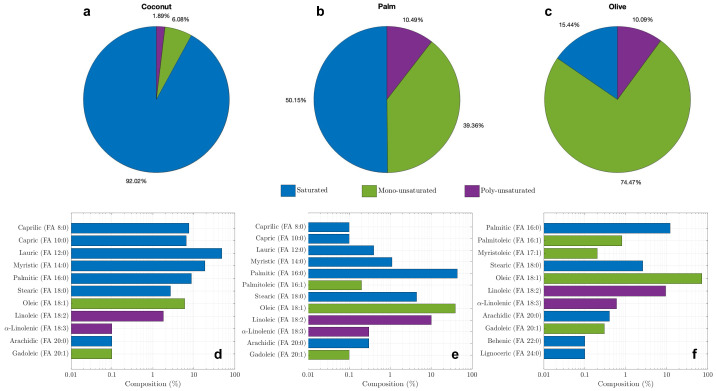
Compositions of plant oils: saturated (blue), mono-unsaturated (green), and poly-unsaturated (purple) for coconut oil (**a**), palm oil (**b**), and olive oil (**c**); fatty acids breakdown for coconut oil (**d**), palm oil (**e**), and olive oil (**f**) [57,58,59].

Similarly, three different kinds of plant oil compositions are shown in Figure 2 [57,58,59]. Notably, coconut oil contains the largest fraction (92.02%) of saturated fatty acids, whereas olive oil has the lowest fraction (15.44%) of saturated fatty acids among all the three animal fats and the three plant oils. Unlike animal fats, interestingly in these three plant oils, lauric acid (12:0) is the major saturated acid and these also have the largest unsaturated acid of oleic (18:0).

Therefore, based on the properties of each component in bio-PCMs, in this paper, a comprehensive study of different processes has been conducted to tailor the multiphysical properties of the PCM, while addressing the three challenges of the partial processes, limited latent heat, and inconsistent food source, as further research was called to use a consistent source as feedstock [60].

## 2. Materials and Methods

In this study, considering consistent feedstock, a commercially processed bacon fat product, called BaconUp, was chosen to have reliable source with a long shelf life according to the manufacturer. Although animal fat generally contains at least 50% unsaturated fatty acids (mainly oleic acid) that are liquid at room temperature, the bacon fat remained solid due to the presence of saturated fatty acids with higher melting points and associated thermophysical properties.

**Figure 3:**
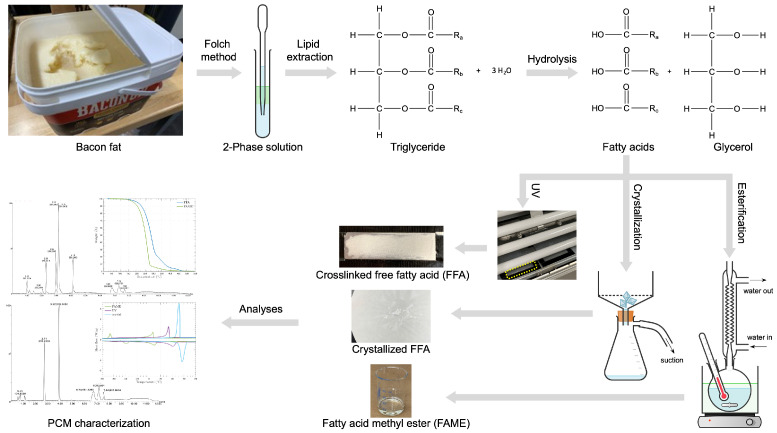
Flow diagram of PCM synthesis process from bacon fat with thermophysical-chemical analyses and characterization.

The upper part of Figure 3 shows that the lipids extracted from the bacon fat were triglycerides, which consist of three glycerol molecules and three fatty acids, collectively known as triacylglycerol, where the fatty acids are bonded to a glycerol backbone.

As detailed in the next sections of the experimental procedures, the lipids were extracted using a modified Folch method, and free fatty acids (FFAs) were produced through hydrolysis. Additionally, as depicted in the lower part of Figure 3, the obtained FFAs were further processed to alter their molecular structures and compositions using three distinct synthesis methods: (1) esterification, (2) UV-crosslinking, and (3) crystallization, resulting in three different types of PCMs. This approach allows for the customization of PCM properties for various thermal energy storage applications.

### 2.1. Bio-PCM Preparation

The Folch method was adapted to extract lipids from animal fats as follows [61]. First, 1 g of animal fat was weighed and placed in a tube. A mixture of chloroform and methanol in a 2:1 ratio was added, with the total volume being 20 times that of the fat sample. The tube was then sealed and shaken for 3-5 min to homogenize the tissue. To ensure sufficient material, two identical samples were prepared. After the fat was dissolved in the solvent, methanol was added, and the tube was shaken again to ensure complete dissolution. The solution was then washed with 4 mL of 0.9% NaCl solution, shaken, and transferred to a beaker. This resulted in a two-phase solution, with methanol and unwanted substances forming the top layer, while chloroform, lipid molecules, and the NaCl solution settled at the bottom. As shown in Figure 3, a pipette was carefully used to separate the lower layer from the upper layer and transfer it to a new tube, ensuring that none of the upper layer was drawn in. Anhydrous sodium sulfate (Na_2_SO_4_) was added to absorb any remaining water molecules, and a small pipette fitted with cotton was used to filter out the Na_2_SO_4_. Finally, a rotary evaporator was employed to evaporate the chloroform, leaving behind the extracted lipid.

A hydrolysis reaction was employed to break down triglycerides into free fatty acids. To prepare a 1.75 M solution (300 mL at 90% *v*/*v*), 29.4 g of potassium hydroxide (KOH) was dissolved in a mixture of 30 mL of deionized (DI) water and 270 mL of ethanol in a flask. After adding 50 g of bacon fat to the solution, the mixture was heated to 70 °C using a magnetic stirrer inside an oil bath on a hot plate for 105 min, until the fat was fully dissolved. To separate the unsaponifiable materials, 200 mL of water and 100 mL of n-hexane were added, followed by double separation using a separatory funnel. The aqueous alcohol phase containing the soap was then acidified with 120 mL of 6N hydrochloric acid (HCl) and transferred to a separatory funnel again to separate the potassium chloride (KCl) and water phase from the hexane and fatty acids. Next, sodium sulfate (Na_2_SO_4_) was added to the mixture to remove any remaining water from the free fatty acids and hexane solution, and the sodium sulfate was filtered out. Finally, the solution was transferred to a flask, and the hexane was evaporated using a rotary evaporator, leaving behind the free fatty acids.

Fatty acid methyl esters (FAME) were synthesized from free fatty acids (FFAs) based on the previous method [62]. First, 10 g of FFAs were added to a flask, and a reagent solution was prepared by mixing 50 mL of methanol with 12.5 mL of hydrochloric acid (HCl). To the flask, 37.5 mL of methanol, 7.5 mL of the reagent, and 7.5 mL of toluene were added, and the mixture was manually stirred. A magnetic stir bar was then placed inside the flask, which was set on a hot plate at 65 °C, and the mixture was refluxed for 1.5 h. After refluxing, 75 mL of hexane and 50 mL of DI water were added to the flask and manually stirred. The mixture was then transferred to a separatory funnel, which was inverted and shaken to release hexane vapor through the valve. After the layers separated, the bottom layer was discarded, leaving only the upper layer containing hexane and FAME. To ensure the purity of the FAME, a small portion of the hexane-FAME layer was drained during separation to remove any residual contaminants from the lower layer. Sodium sulfate (Na_2_SO_4_) was then added to the upper layer to absorb any remaining water molecules. Finally, after filtering the salt using a Buchner funnel with filter paper of medium flow rate and 8 µm particle retention, the FAME was left in a fume hood overnight to allow the hexane to evaporate.

After 1 g of FFAs and 5 mL of methanol were added to a flask, it was placed on a hot plate with a magnetic stir bar inside and heated under reflux conditions at 50 °C for 1 h, with a stirring speed of 200 rpm, as shown in the lower right of Figure 3. The temperature was chosen to stay below methanol’s boiling point of around 64.7 °C. Once the FFAs were completely dissolved in the methanol, the mixture was transferred to a beaker and allowed to cool at room temperature (22 °C) for 1 h. The sample was then placed in a freezer, where it was cooled at a rate of 0.1 °C per minute until reaching a target temperature of −20 °C. This 44 °C temperature drop from room temperature (22 °C) to the target temperature (−20 °C) took approximately 7 h and 20 min. After cooling, the solution was filtered using a filter paper on a Buchner funnel with gentle suction. The material remaining on the filter paper was then placed in a desiccator for a few hours to allow the methanol to evaporate and the solution to dry.

FFAs were subjected to UV light exposure in an accelerated weathering tester (Q-LAB QUV). To prepare the sample, an aluminum plate was partially covered with UV-resistant Black Gorilla tape at the corners, while FFAs of 0.235 g were evenly spread in the center. The plate was then heated to 75 °C on a hot plate, and the FFAs were evenly distributed using a small spatula to create a uniform layer. The sample was then placed in the weathering tester, where it was exposed to UV light simulating sunlight at a wavelength of 340 nm for 24 h. The temperature inside the QUV chamber reached and maintained 45 °C throughout the exposure due to the heat generated by the UV lamps.

### 2.2. Characterization of Bio-PCMs

A chromatography system (Waters^*TM*^ ACQUITY Arc H-Class UPLC) was combined with a high-resolution quadrupole time-of-flight mass spectrometer (Waters^*TM*^ Xevo G2-XS Q-ToF, Milford, MA, USA). The mobile phase employed blank solvents composed of Water/Acetonitrile/2-Propanol in a 2:4:4 (*v*/*v*/*v*) ratio. Using a CSH Acquity UPLC column (C18), the mass spectrum analysis was performed with the two solvents system: water/acetonitrile (40:60; *v*/*v*) with 10 mM ammonium formate and water/acetonitrile/2-propanol (5:10:85; *v*/*v*/*v*) with 10 mM ammonium formate.

A TA Instruments TGA Q50 was utilized, capable of operating from ambient temperature up to 1000 °C with a controlled heating rate ranging from 0.1 to 100 °C/min. It measures mass changes with a weighing precision of 0.01% and a sensitivity of 0.1 µg, accommodating samples up to 1 g. In this experiment, 0.025 g of FFAs and 0.043 g of FAME were heated at a rate of 10 °C/min from room temperature to 550 °C, a temperature safely below the softening point of the Tzero aluminum pan used for the analysis.

A TA Instruments Discovery DSC 25 was employed, offering a temperature range from −90 °C to 550 °C with a refrigeration unit. Prior to analyzing the samples, baseline calibration was carried out using high-purity Indium as the reference material and a nitrogen gas flow rate of ±5 mL/min. Following calibration, Tzero aluminum pans with Tzero Hermetic lids were used to prepare the specimens, particularly for volatile materials. The evaluation procedure included the following steps: (i) the temperature was increased to 90 °C to ensure that the pre-melted samples were uniformly distributed within the DSC pan, (ii) an isothermal condition at 90 °C was maintained for 5 min to achieve equilibrium, (iii) the samples were then cooled at a rate of 5 °C/min until reaching −90 °C, (iv) an isothermal condition was maintained at −90 °C for 5 min, (v) the samples were reheated to 90 °C at a rate of 5 °C/min, and (vi) a final isothermal stage at 90 °C was held for 5 min. After the evaluation was completed, the samples were cooled to 40 °C, at which point the equipment was set to idle.

## 3. Results and Discussion

### 3.1. Molecular Analysis

**Figure 4:**
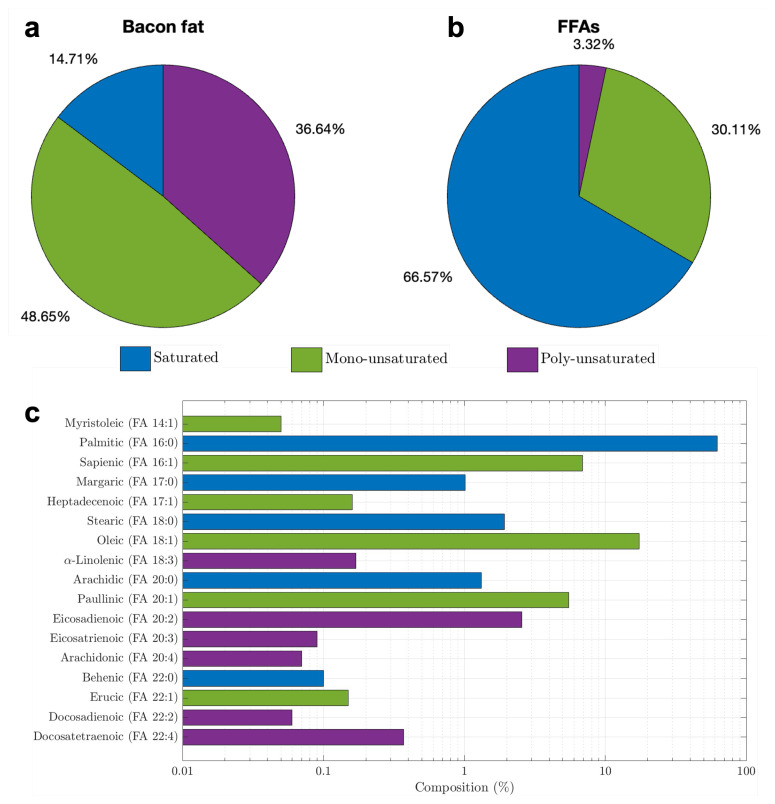
Composition analyses in terms of saturated (blue color), mono-unsaturated (green color), and poly-unsaturated (purple color) for (**a**) Bacon fat, (**b**) FFAs, and (**c**) individual acids in the FFAs.

The UPLC-MS data, analyzed with the LIPID MAPS library, revealed the composition of the bacon fat and FFAs. In the bacon fat (Figure 4a), 14.71% of the triglyceride molecules were saturated, while 85.29% were unsaturated, with 48.65% being mono-unsaturated and 36.64% poly-unsaturated. For the FFAs (Figure 4b), 66.56% were saturated, and 33.44% were unsaturated, including 30.11% mono-unsaturated and approximately 3.32% poly-unsaturated.

Triglycerides are classified based on the presence of carbon-carbon double bonds (C=C) in their fatty acid chains. If none of the three fatty acid chains contain a C=C bond, the triglyceride is fully saturated. If one C=C bond is present in any of the chains, the triglyceride is mono-unsaturated, regardless of whether one, two, or all three chains have a C=C bond. Triglycerides with more than one C=C bond in any chain are classified as poly-unsaturated.

Specifically, the fatty acid composition of the hydrolyzed FFAs was analyzed in terms of saturated, mono-unsaturated, and poly-unsaturated fatty acids (Figure 4c). Notably, 62.20% of the FFAs were palmitic acid, a saturated fatty acid. Other saturated fatty acids, such as stearic acid (1.92%), margaric acid (1%), arachidic acid (1.3%), and behenic acid (0.10%), were present in smaller amounts. Oleic acid, the most abundant unsaturated fatty acid at 17.37%, contributed to the FFAs being solid at room temperature due to the high proportion of saturated fatty acids.

Additionally, the number of carbons in the hydrocarbon chains of the FFAs ranged from 14 to 22, with up to four C=C bonds in the unsaturated molecules. In contrast, triglycerides had longer carbon chains (42 to 59 carbons) and up to 13 C=C bonds. The hydrolysis process reduced these large triglyceride molecules into smaller FFA molecules, increasing the relative concentration of saturated fatty acids, which was advantageous for this study.

Finally, the mass spectrum of the crystallized FFAs displayed fewer peaks, indicating the removal of certain components. The two prominent peaks appeared at 2.71 and 3.92 min, with mass-to-charge ratios of 255.23 and 283.26, respectively. These peaks corresponded to palmitic acid (FA 16:0) and stearic acid (FA 18:0), as identified from the FFA database.

### 3.2. Thermal Stability

**Figure 5:**
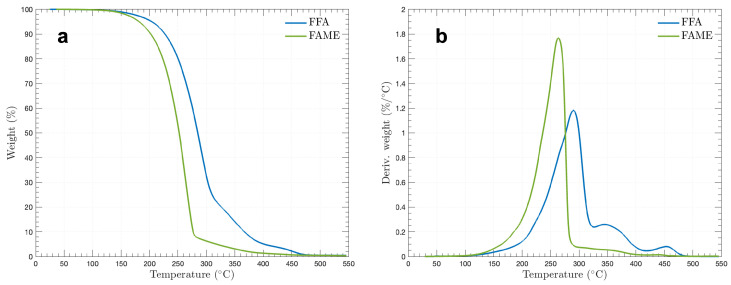
Thermogravimetric analysis (**a**) and differential thermogravimetric analysis (**b**) for FFA and FAME.

Thermogravimetric analysis (TGA) was used to assess the thermal stability of the PCMs and to analyze the proportions of their volatile components by tracking weight changes over time as the temperature rises. As illustrated in Figure 5, 0.025 g of free fatty acids (FFAs) and 0.043 g of fatty acid methyl ester (FAME) were heated at a rate of 10 °C/min from room temperature to 550 °C, remaining below the softening point of the Tzero aluminum pan used. The absence of significant weight loss up to about 110 °C suggests that neither the FFAs nor the FAME contained moisture after processing (see Figure 3).

Figure 5a indicates that FFAs experienced notable weight loss starting at 227.83 °C, with a continuous decline until around 307 °C, where the rate of weight loss began to slow. The sample then entered a third phase of mass loss at 380.13 °C, with 94.18% of the sample evaporated by this point. Complete degradation did not occur until 480 °C, and the absence of multiple weight loss stages suggests the sample was homogeneous, lacking components with varied degradation temperatures. In contrast, composites with multiple components typically show a plateau before experiencing further mass loss. Additionally, Figure 5 shows that FAME began to lose weight at 276.70 °C, where 91.80% of the mass was lost. The sample continued to lose mass at a reduced rate until fully combusted at about 500 °C.

The observed weight loss behavior is likely influenced by the predominant palmitic acid in the FFAs or palmitic acid methyl ester in the FAME. The constituent acids in the FFAs generally have higher melting temperatures ($T_{m}$) and boiling temperatures ($T_{b}$) compared to those in FAME, as summarized in Table 4 [63,64]. Melting properties are typically determined by the chemical structures of the compounds, including chain length and bond configurations. Compounds with longer hydrocarbon chains, such as those in both FFAs and FAME, exhibit higher $T_{m}$, whereas FAME with shorter chains tend to have lower $T_{m}$ relative to their corresponding FFAs [64]. Palmitic and stearic acid methyl esters are solid at room temperature due to their straight-chain structures and relatively high $T_{m}$, while oleic acid methyl ester remains liquid at room temperature due to its bent structure and lower $T_{m}$ [65].

### 3.3. Thermophysical Properties of the Bio-PCMs

**Figure 6:**
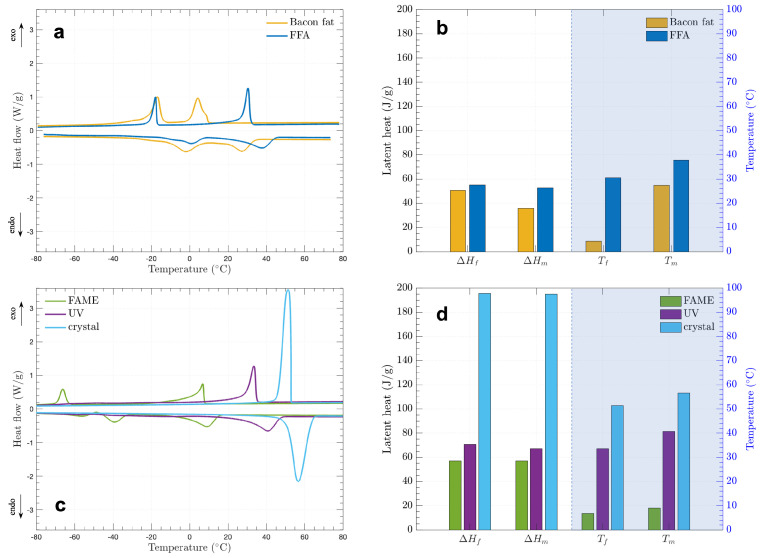
Differential scanning calorimetry for PCMs: (**a**) heat flow with temperature for bacon fat and FFA, (**b**) hydrolysis effect on latent heat and phase transition temperature, (**c**) heat flow with temperature for FAME, UV, and crystal, (**d**) latent heat change and phase transition temperature with esterification, UV-crosslinking, and crystallization.

The differential scanning calorimetry (DSC) analyses for all the materials in this study are presented in Figure 6a,c. In these figures, exothermic peaks, which indicate heat release, are shown as upward peaks, while endothermic peaks, which signify heat absorption, are displayed as downward peaks. The upward curve represents a temperature decrease from 80 °C to −80 °C, while the downward curve shows a temperature increase from −80 °C to 80 °C. This temperature range was selected to prevent any material degradation based on TGA results. Furthermore, the phase change properties for all measured materials are listed in Table 5. Figure 6b,d illustrate the latent heats in conjunction with phase transition temperatures around 10 °C or higher, which is relevant for potential applications in ambient conditions.

Figure 6a indicates the phase change characteristics of bacon fat compared to free fatty acids (FFAs), processed as shown in the upper flow section of Figure 3. Both bacon fat and FFAs exhibit two peaks for each exothermic and endothermic process due to the presence of saturated, mono-unsaturated, and poly-unsaturated fatty acids [29].

The area under the curves represents the latent heat values. For bacon fat, the freezing latent heat ($\Delta H_{f}$) was 50.61 J/g at a freezing temperature ($T_{f}$) of 4.23 °C for the right-hand peak and 62.97 J/g at $T_{f}$ = −16.48 °C for the left-hand peak in the exothermic process. For the endothermic peaks, the melting latent heat ($\Delta H_{m}$) was 42.11 J/g at a melting temperature ($T_{m}$) of −2.14 °C for the left-hand peak and 35.63 J/g at $T_{m}$ = 27.35 °C for the right-hand peak. These results indicate that the raw bacon fat is not ideal for phase change material (PCM) applications in thermal storage, due to its low latent heat and phase transition temperatures.

Following the hydrolysis reaction, a notable shift in phase transition temperature was observed. In Figure 6a, the right-hand peak for bacon fat’s exothermic transition moved from $T_{f}$ = 4.23 °C to $T_{f}$ = 30.45 °C in the FFAs. This shift is attributed to the higher saturated fatty acid content in FFAs (66.57%) compared to bacon fat (14.71%), as shown in Figure 4. Additionally, the exothermic heat flow in the right-hand peak for FFAs increased to 1.24 W/g from 0.95 W/g in bacon fat, while the heat flow in the left-hand peak was similar but narrower for FFAs. For the endothermic peaks, the left-hand side temperature of the FFAs was similar to that of bacon fat, though with a lower $\Delta H_{f}$ value. The melting properties further supported the hypothesis that FFAs contain more saturated and fewer unsaturated fatty acids. Specifically, the left-hand endothermic peak at 0.79 °C had a lower $\Delta H_{m}$, while the right-hand peak at 37.78 °C had a higher $\Delta H_{m}$ of 52.73 J/g. Despite the increased latent heat at higher phase transition temperatures, the values of $\Delta H_{f}$ and $\Delta H_{m}$ were still insufficient for PCM applications, necessitating further synthesis.

Esterification was explored as an additional synthesis method for the FFAs. Sarı et al. synthesized esters with myristic, palmitic, and stearic acids and showed latent heat of 149–185 J/g, while esters have also other advantages over fatty acids, such as less odor, lower corrosivity, and a wider phase transition temperature [66,67,68]. Figure 6c shows that esterification reduced the freezing temperatures ($T_{f}$) to 6.79 °C for the left-hand peak and −66.19 °C for the right-hand peak, and melting temperatures ($T_{m}$) to −39.40 °C for the left-hand peak and 9.00 °C for the right-hand peak. Although there was a slight improvement in latent heat values, both $T_{f}$ and $T_{m}$ decreased significantly, making FAME more suitable for chilling and packaging applications rather than thermal storage.

Another process is irradiating ultraviolet (UV) rays which were previously used to form crosslinked structures about acrylated soybean oil-based PCMs to prevent leakage, as UV-crosslinking lengthens the chains of fatty acids, resulting in higher the both melting and freezing temperatures [69]. As shown in Figure 6c,d, exposing FFAs to UV rays for 24 h led to the disappearance of one peak in both exothermic and endothermic curves. The UV-crosslinked FFAs showed an improved $\Delta H_{f}$ of 70.94 J/g with $T_{f}$ = 33.53 °C and $\Delta H_{m}$ of 67.22 J/g with $T_{m}$ = 33.53 °C.

Lastly, while esterification and UV treatments did not significantly enhance latent heat, the crystallization process was used to separate saturated fatty acids from unsaturated fatty acids of animal fats or plant oils. Typically, when fatty acids are cooled, they begin to aggregate, forming clusters that eventually lead to a crystallized structure. This is driven by the self-assembly of long-chain alkyl groups, influencing thermodynamics and the kinetic path to the crystalline state [70]. For such crystallization, the solution of fatty acids dissolved in a solvent needs to be supersaturated: when cooling down a saturated solution from a specific temperature, the solution becomes supersaturated as solubility decreases with temperature. Furthermore, a slower cooling rate of crystallization induces larger size of clusters [71], and the crystallized fatty acids mainly consist of saturated acids having straight hydrocarbon chain structures [56,64]. Thus, the crystallization process produced noticeable changes in heat flow peaks and latent heat values, similar to UV-crosslinked FFAs, as it removed unsaturated fatty acids. The crystallized FFAs exhibited significant improvements, with $\Delta H_{f}$ = 195.55 J/g and $\Delta H_{m}$ = 195.19 J/g, compared to $\Delta H_{f}$ = 55.05 J/g and $\Delta H_{m}$ = 52.73 J/g for the FFAs. This increase represents a 3.6-fold and 3.7-fold improvement in freezing and melting latent heats, respectively. The crystallized FFAs predominantly contained palmitic acid (FA 16:0) and stearic acid (FA 18:0), both of which are saturated fatty acids. The measured latent heats were close to the reported values for these acids.

In addition to enhanced latent heats, Figure 6d and Table 5 show that crystallization also improved phase transition temperatures, with $T_{f}$ rising from 30.45 °C to 51.45 °C and $T_{m}$ increasing from 37.78 °C to 56.61 °C. These temperatures are consistent with the reported ranges for palmitic and stearic acids, indicating that the crystallized FFAs primarily consist of these acids with their straight-chain structures.

Finally, unlike the broad peaks observed in palm oil-based PCMs, the crystallized FFAs exhibited sharp and narrow peaks in both freezing and melting characteristics, making them favorable for thermal storage applications. These FFAs can be activated to absorb or release heat at specific temperatures, and mixing them with other PCMs could create a eutectic PCM with adjustable phase transition temperatures based on component concentration. Therefore, the combination of palmitic and stearic acids in the crystallized FFAs makes them a promising candidate for animal fat-based PCMs.

## 4. Future Work on Bio-PCM Development

The melting temperature and latent heat of bio-PCMs are of great significance to their applications. In our present work, bio-PCMs exhibit great potentials to achieve the similar or higher latent heat than the commercial paraffin-based PCMs through separation and purification of FFA components. The test results of the three bio-PCMs show that the esterification significantly decreased the phase transition temperatures, but the latent heat only increased slightly, the UV-treatment moderately raised both the phase transition temperature and latent heat, while the crystallization process provided the most desirable results. Following this work, the future research focuses on mass production of bio-PCMs with enhanced latent heat and tailorable melting temperature with the following highlights:

1. For large-scale manufacturing, the fatty acids can be separated into individual components in Table 3 which typically exhibit higher latent heats than the eutectic alternatives, and the cost-benefit analysis will be conducted to optimize the yield.
2. The unsaturated fatty acids can be converted to saturated fatty acids by hydrogenation as the saturated ones commonly exhibit a higher latent heat. Different saturation levels provide different melting temperature and latent heat, which can be used to design bio-PCMs for specific applications.
3. The saturated fatty acids can be crystallized into a highly structured solid, which may significantly increase the latent heat. The durability and lifetime of the bio-PCMs will be investigated to assure the long-term performance with environmental impact.
4. Multiscale modeling and characterization of the molecular and atomic structure changing with temperature can predict and optimize the PCM synthesis process. Our recently developed singum model [72] can predict the stiffness changing with temperature. It can be extended to evaluate the latent heat with the nanostructure of the bio-PCM, and thus provide a guideline for material design.
5. Since sources of animal fats and plant oils are still various and scattered at the same time [19], it is necessary to explore more research with consistent source from plants or animals.
6. As effective thermal storage needs to have high thermal conductivity as well as high latent heat, novel materials or synthesis processes will be explored to overcome the trade-off relationship between those quantities for practical usage of sustainable bio-based PCMs.

Overall, the success of this technology to transform fatty acids of food wastes into bio-PCMs will not only recycle animal fats for a higher-value product at the end of their life cycles but also enable new applications in thermal management with the affordable, sustainable PCM supplies.

## 5. Conclusions

Bio-PCMs are attractive candidates for passive heat storage applications which doesn’t require any additional energy and for sustainable development goals in terms of hunger reduction, affordable and clean energy, responsible consumption and production, and climate action. In this work, a comprehensive material process was demonstrated to convert animal fats into phase change materials by achieving sufficient latent heat and phase transition temperature for thermal storage applications. Using the bacon fats containing nearly 15% saturated fatty acids, hydrolysis was applied to break the triglyceride molecules and to obtain FFAs having 62% palmitic acids.

The three further processes were developed to engineer phase change properties of the FFAs: (1) the esterification reduced phase transition temperatures by modifying its hydrocarbon chain structure; (2) the UV-crosslinking increased the both latent heats and phase transition temperatures by about 10% due to the longer chain length; (3) the crystallization significantly enhanced the latent heats by about 3.5 times with the highest phase transition temperatures via separating the saturated fatty acid from the unsaturated, resulting in the only two components of palmitic acid and stearic acid.

Such achievements with the crystallized FFAs show that the meat-based products containing palmitic and steric acids can be used as practical PCMs through the proposed processes. As characterized in this work, the latent heats were not only about 195 J/g but also singular at the corresponding phase transition temperatures, consequently being beneficial to be applied for certain temperatures or to be even more tunable by mixing with others. Therefore, bacons or meat-based products would be attractive sources to show wide ranges of the both phase transition temperature (e.g., $T_{m}$ = −39.40 − 56.61 °C) and latent heat (e.g., $\Delta H_{m}$ = 35.63 − 195.19 J/g), comparable to commercial PCMs, as well as to contribute to reducing GHGs in line with SDGs.

## Figures and Tables

**Table 1:** Contribution of bio-PCMs to the sustainable development goals (SDGs).

SDG	Contribution of PCMs	Ref
2. Zero Hunger	Improving crop quality and productivity	[15]
7. Affordable and Clean Energy	Incorporating passive into active thermal systems for space cooling	[11]
	Lowering the cost of heat storage and transportation	[17]
	65% reduced auxiliary heater load	[22]
	53.7% longer heat transfer duration and 58.9% reduced annual energy cost for electrical heater	[23]
12. Responsible Consumption and Production	Reducing food wastes for the passive thermal method	[21]
13. Climate Action	Reducing 8.03–10.95 t CO_2_ equivalent per year per hectare by decreasing operation of root zone heating systems	[16]
	Decreasing CO_2_ emissions up to 93% by mobilized thermal energy storage	[24]

**Table 2:** Thermal properties of bio-PCMs from various sources.

Source	PCM	$T_{m}$ °C)	$\Delta H_{m}$ (J/g)	κ (W/mK)	Ref
Italian sausage	Filtered pork fat	0	43.3	0.21	[27]
		32	21.3		
Local market and restaurant	Mixture of pork fat with burnt oil	-	-	0.19	[28]
Animal parts from a slaughterhouse	Mostly pig and chicken paste	2	5.67	-	[29]
		25	23.27		
Non-edible fatty pig and chicken parts	PA-SA mixture	53.4–55.2	157–183	-	[30]
	DHSA	93.8	161		
	DHSA salts	160–231.6	76–236		
Beef tallow from market	Filtered beef fat	37.4	101.05	-	[31]
Soya Group	Coconut oil	24	115.31	–	[32]
Bakery	Palm oil	3.29	33.03	-	[33]
		33.82	13.68		
Pacific Interlink SDNBHD	Hydrogenated palm kernel fat	26.53	74.35	0.20	[34]

**Table 3:** Structures and properties of fatty acids in bio-PCMs.

Saturated	Formula	$T_{m}$ (°C)	$\Delta H_{m}$ (J/g)	κ (W/mK)	Ref
(8:0) Caprylic	CH_3_(CH_2_)_6_COOH	15.4–16.1	142.6–158.4	–	[38,39,40,41]
(10:0) Capric	CH_3_(CH_2_)_8_COOH	29.6–31.5	139.8–155.5	0.15	[42,43]
(12:0) Lauric	CH_3_(CH_2_)_10_COOH	42.6–44.3	176.6–179.9	0.15	[44,45]
(14:0) Myristic	CH_3_(CH_2_)_12_COOH	49.0–63.3	178.1–210.7	0.15	[44,46]
(16:0) Palmitic	CH_3_(CH_2_)_14_COOH	58.9–69.4	164.8–420.0	0.16	[44,47]
(18:0) Stearic	CH_3_(CH_2_)_16_COOH	55.0–77.6	185.4–259.0	0.17	[44,47]
(20:0) Arachidic	CH_3_(CH_2_)_18_COOH	75.2	257.4	–	[48]
(22:0) Behenic	CH_3_(CH_2_)_20_COOH	69–88	232	–	[49]
**Unsaturated**	**Formula**	$T_{m}$ **(°C)**	$\Delta H_{m}$ **(J/g)**	κ **(W/mK)**	**Ref**
(16:1) Palmitoleic	CH_3_(CH_2_)_5_CH= CH(CH_2_)_7_COOH	2	125.2	-	[50]
(18:1) Oleic	CH_3_(CH_2_)_7_= CH(CH_2_)_7_COOH	13.6	138.1	0.10	[51]
(18:2) Linoleic	CH_3_(CH_2_)_4_CH= CHCH_2_CH= CH(CH_2_)_7_COOH	33.6	180.5	-	[51]
(18:3) α-Linolenic	CH_3_(CH_2_CH=CH)_3_ (CH_2_)_7_COOH	27.8	164.8	-	[51]

**Table 4:** Selected fatty acids with melting temperature ($T_{m}$) and boiling temperature ($T_{b}$) [63,64].

C:D Ratio	Name	$T_{m}$ (°C)		$T_{b}$ (°C/10 mmHg)	
		FFA	FAME	FFA	FAME
FA 16:0	Palmitic	62.5–64.0	28.5–30.5	212	184
FA 18:0	Steric	68.8–71.2	37.7–39.1	227	205
FA 18:1	Oleic	13.2–16.3	−20.2–19.6	223	201

**Table 5:** Phase change properties of bacon fat, free fatty acid (FFA), fatty acid methyl ester (FAME), UV-crosslinked FFA (UV), and crystallized FFA (crystal): freezing temperature ($T_{f}$), freezing latent heat ($\Delta H_{f}$), melting temperature ($T_{m}$), and melting latent heat ($\Delta H_{m}$).

PCM	Freezing Properties			Melting Properties	
	$T_{f}$ **(°C)**	$\Delta H_{f}$ **(J/g)**		$T_{m}$ **(°C)**	$\Delta H_{m}$ **(J/g)**
Bacon fat	4.23	50.61		27.35	35.63
	−16.48	62.97		−2.14	42.11
FFA	30.45	55.05		37.78	52.73
	−17.82	27.08		0.79	25.89
FAME	6.79	57.08		9.00	57.05
	−66.19	22.40		−39.40	26.54
UV	33.53	70.94		40.80	67.22
crystal	51.42	195.55		56.61	195.19

## Data Availability

Data will be made available on request.
